# Histoplasmosis Outbreak Associated with the Renovation of an Old House — Quebec, Canada, 2013

**Published:** 2014-01-03

On May 19, 2013, a consulting physician contacted the Laurentian Regional Department of Public Health (Direction de santé publique des Laurentides [DSP]) in Quebec, Canada, to report that two masons employed by the same company to do demolition work were experiencing cough and dyspnea accompanied by fever. Other workers also were said to be ill. DSP initiated a joint infectious disease, environmental health, and occupational health investigation to determine the extent and cause of the outbreak. The investigation identified 14 persons with respiratory symptoms among 30 potentially exposed persons. A strong correlation was found between exposure to demolition dust containing bat or bird droppings and a diagnosis of histoplasmosis. Temporary suspension of construction work at the demolition site in Saint-Eustache, Quebec, northwest from Montreal, and transport of the old masonry elements to a secure site for burial were ordered, and information about the disease was provided to workers and residents. To prevent future outbreaks, recommendations included disinfection of any contaminated material, disposal of waste material with proper control of aerosolized dust, and mandatory use of personal protective equipment such as gloves, protective clothing, and adequate respirators.

Histoplasmosis is an infectious disease caused by inhalation of spores produced by the fungus *Histoplasma capsulatum* (HC) (1,2). The organism can be excreted by bats and birds in their droppings and can persist in the environment for several years (3). Pulmonary infection sometimes causes symptoms typical of pneumonia (e.g., dyspnea, fever, and thoracic pain). The incubation period varies ranges from 7 to 21 days. Renovation of old houses that have sheltered colonies of bats has been associated with histoplasmosis resulting from worker exposure to aerosolized spores of the fungus (4–6). Disseminated histoplasmosis is a rare form of the infection that can be fatal, even if properly treated.

On May 19, 2013, a consulting physician contacted DSP to report that two masons employed by the same company were experiencing cough and dyspnea accompanied by fever. Other workers were also reported to be sick. A joint infectious disease, environmental health, and occupational health investigation was initiated by DSP. The objectives of the investigation were to describe the demolition work, the workers, and other persons involved, and the medical history of persons who became ill, to determine the extent and cause of the outbreak.

Initial questioning revealed that the two workers became ill 48 hours earlier. Because of the severity of the symptoms, both patients were referred to the emergency department of a Montreal tertiary-care center. One of the two patients was hospitalized. Further investigation revealed that during May 18–20, 2013, six masons were evaluated in the emergency department for similar symptoms, and two were hospitalized. All the masons had recently carried out demolition of the exterior walls of a century-old brick house and had seen a large quantity of dried bird or bat droppings behind the bricks. The demolition work was reported to have caused a cloud of dust in the immediate environment. Given the history of exposure to droppings, the diagnosis of histoplasmosis was considered.

The investigation led to the identification and questioning of the 30 persons believed to have been exposed to HC from work-site debris during April 29–May 14, 2013. Those 30 included 21 men and nine women, with a mean age of 39 years (median: 30.8 years, range: 16–77 years). A standardized questionnaire was used to record symptoms and determine potential exposures. Half of the exposed person were workers: six masons who demolished the brick walls, four bricklayers, one debris sorter working for a container company from outside the Laurentian region who picked up the demolition debris and transported it to a sorting site away from the demolition site, two other debris sorters from the same company who cleaned the bricks, and two metal workers from a third company who carried out repairs to the roof eaves. The other 15 persons included the homeowner and his wife, who lived on the ground floor of the house, and two tenants living upstairs; three visitors who walked around on the site for 10–90 minutes; and eight neighbors.

Of these 30 persons, 14 experienced respiratory symptoms: six masons, three debris sorters, the two residents on the ground floor, the two neighbors whose bedroom faced the demolition site, and one of the visitors to the site (Table). These 14 persons consulted a physician. Two workers were hospitalized. Symptoms began to appear during May 2–17, with a peak occurring May 13–17 (Figure). In order of frequency, the symptoms were dyspnea (100%), chills (86%), headaches (86%), sweating (79%), chest pain (79%), asthenia (79%), fever (71%), cough (71%), myalgia (57%), nausea (43%), diarrhea (36%), erythema (29%), abdominal pain (14%), and vomiting (14%). The average duration of respiratory symptoms was 12.6 days (median: 13.5 days; range: 5–20 days). All the symptomatic persons recovered without any specific treatment for histoplasmosis.

A clinical case of histoplasmosis was defined as the presentation of at least four of the following symptoms: dyspnea, chest pain, cough, fever, chills, sweating, asthenia, or myalgia, with onset during April 30–May 19, 2013, in a person exposed to the demolition site or involved in the handling of demolition debris during April 29–May 14, 2013. A confirmed case was defined as a case meeting the clinical case definition plus detection of HC antigen in a serum or urine specimen. All of the 14 persons who had respiratory symptoms met at least the clinical case definition. Hospitalized patients underwent radiologic investigation, in conjunction with blood and microbiologic analysis, to rule out other viral, bacterial, or fungal infections, including legionellosis and tuberculosis.

A diagnosis of histoplasmosis was confirmed for the two hospitalized masons through a positive serum and a positive urinary HC antigen test. The diagnosis for the two debris sorters was confirmed by a urinary HC antigen test. Five of the other 11 workers received a clinical diagnosis of histoplasmosis resulting from exposure to the same material as the confirmed cases, the presence of compatible clinical manifestations and chest radiographs demonstrating abnormalities. Among the 15 residents, visitors, and neighbors, the illnesses of five were considered clinical cases of histoplasmosis.

Exposure was categorized as high in persons who directly manipulated contaminated material during the demolition, transportation, or debris removal, and in persons who lived in the house during the renovation. If not present during those activities, persons were considered to have experienced low exposure.

Among the 13 persons categorized as having been highly exposed, 11 experienced symptoms, compared with three of 17 persons with a low level of exposure (relative risk = 4.8, 95% confidence interval = 1.7–13.7) (Table). Simply being present during the demolition (April 29–May 1) was also strongly associated with infection. Of 23 persons present, 14 experienced symptoms, compared with none of the seven persons exposed after demolition (relative risk = ∞, 95% confidence interval = undetermined; p<0.005).

The recommendations made by DSP consisted of temporarily suspending any further construction work and informing the workers and the residents about the disease. The risk for additional contamination from the house’s environment was assessed. The old bricks from the demolition debris were contained and buried underground at a secure site. The debris around the house was removed by workers before involvement of DSP. The house’s surroundings were washed by heavy rains during the following days. The Laurentian Regional Occupational Health and Safety Commission also made recommendations to the employers concerning similar work in the future: communicate health risks to workers and insist on preventive measures, particularly the constant use of a respirator. Although the masons were provided with respirators, they wore them intermittently because of the hot weather; respirators were not made available for the three debris sorters.

## Reported by

*André Allard, FRCPC, Denise Décarie, MD, Jean-Luc Grenier, MD, Marie-Claude Lacombe, MD, Francine Levac, MD, Laurentian Regional Dept of Public Health, Saint-Jérôme, Québec, Canada.* ***Corresponding contributor:*** *Jean-Luc Grenier, jean-luc_grenier@ssss.gouv.qc.ca, 450-436-8622 ext. 70520.*

## Editorial Note

A wide range of activities have been associated with histoplasmosis outbreaks: construction, maintenance, renovation, excavation (4–6); caving (7); school activities or day camp (8); search for treasure (9); and agricultural activities (10), among others. The common variable inherent in these activities is the exposure to bird or bat droppings (1) or contaminated soil.

When buildings, particularly old houses, have previously sheltered colonies of bats or birds, appropriate measures should be taken before starting renovation work to protect the health of persons in and around the area.

In this investigation, the confirmation of a diagnosis of histoplasmosis for debris sorters who did not work at the demolition site but handled contaminated materials away from the site demonstrates that the radius of exposure might be greater than expected. As a result, protective measures should be recommended to all workers who might be exposed to contaminated material.

The findings in this report are subject to at least two limitations. First, a conservative approach to risk assessment was adopted by including persons such as residents of the house in the high exposure scenario, and by including clinical cases that could be related to an etiology other than histoplasmosis. Second, the small number of persons involved in this outbreak limits the power of analysis and the conclusions that can be drawn from the investigation. Moreover, the even smaller number of symptomatic persons who were tested for HC antigen reduces the specificity of the diagnosis. Despite these limitations, the high relative risk shows a strong correlation between demolition dust exposure and the onset of disease.

What is already known on this topic ?

Histoplasmosis outbreaks can occur when demolition work produces dust containing bird or bat droppings.

What is added by this report?

During the renovation of an old house in Quebec, Canada, 14 of 30 workers and residents exposed to dust from bird or bat droppings experienced respiratory symptoms consistent with histoplasmosis. Of the four persons whose infection was laboratory-confirmed, two were hospitalized. Illness was highly correlated with exposure to dust during demolition of the exterior walls, and with the handling of contaminated debris away from the work site.

What are the implications for public health practice?

Employers need to provide the appropriate protective equipment and reinforce to employees the necessity of applying protective measures during demolition work, including when handling debris away from the work site.

This outbreak highlights the importance for employers to understand the health risks associated with renovation of old houses in areas where bats or birds roost. Employers should also be made aware of the recommended health measures for their workers, such as wearing a respirator (1).

## Figures and Tables

**FIGURE:**
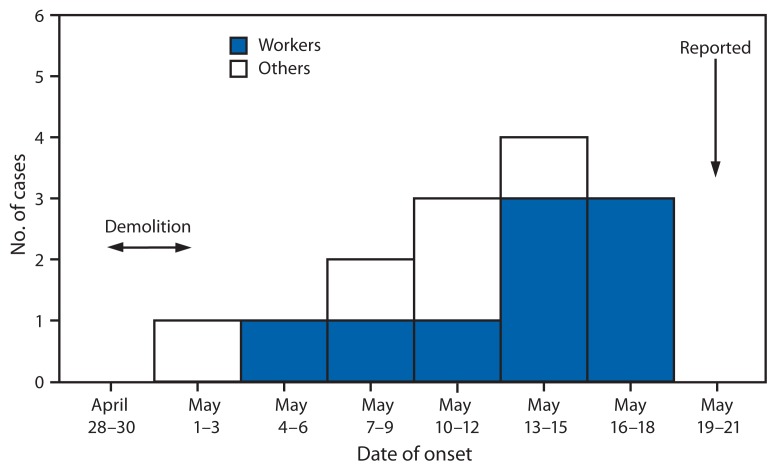
Number of histoplasmosis cases associated with the renovation of an old house, by worker status and date of illness onset — Quebec, Canada, May 1–18, 2013

**TABLE:** Number of histoplasmosis cases associated with the renovation of an old house, by exposure level, hospitalization status, and worker/resident status — Quebec, Canada, May 1–18, 2013

occupation	Total	Exposure level	Case		Histoplasmosis cases	
	
			Yes	No	Confirmed	Hospitalized
**w** **orkers**	15		9	6	4	2
Masons	6	High	6	0	2	2
Bricklayers	4	Low	0	4		
Debris sorters	3	High	3	0	2	
Metal workers	2	Low	0	2		
**o** **thers**	15		5	10	0	0
Residents	4	High	2	2		
Neighbors	8	Low	2	6		
Visitors	3	Low	1	2		
**t** **otal**	**30**		**14**	**16**	**4**	**2**
